# Unhelpful microbes inactivate diabetes drug

**DOI:** 10.1038/s43856-021-00068-2

**Published:** 2022-01-06

**Authors:** Ben Abbott

**Affiliations:** Communications Medicine, https://www.nature.com/commsmed

## Abstract

Drugs can modify the microbiome and, reciprocally, the microbiome can impact drug efficacy. A recent study in *Nature* identifies a potential mechanism through which oral and gut bacteria selectively inhibit the antidiabetic drug acarbose.

**Figure:**
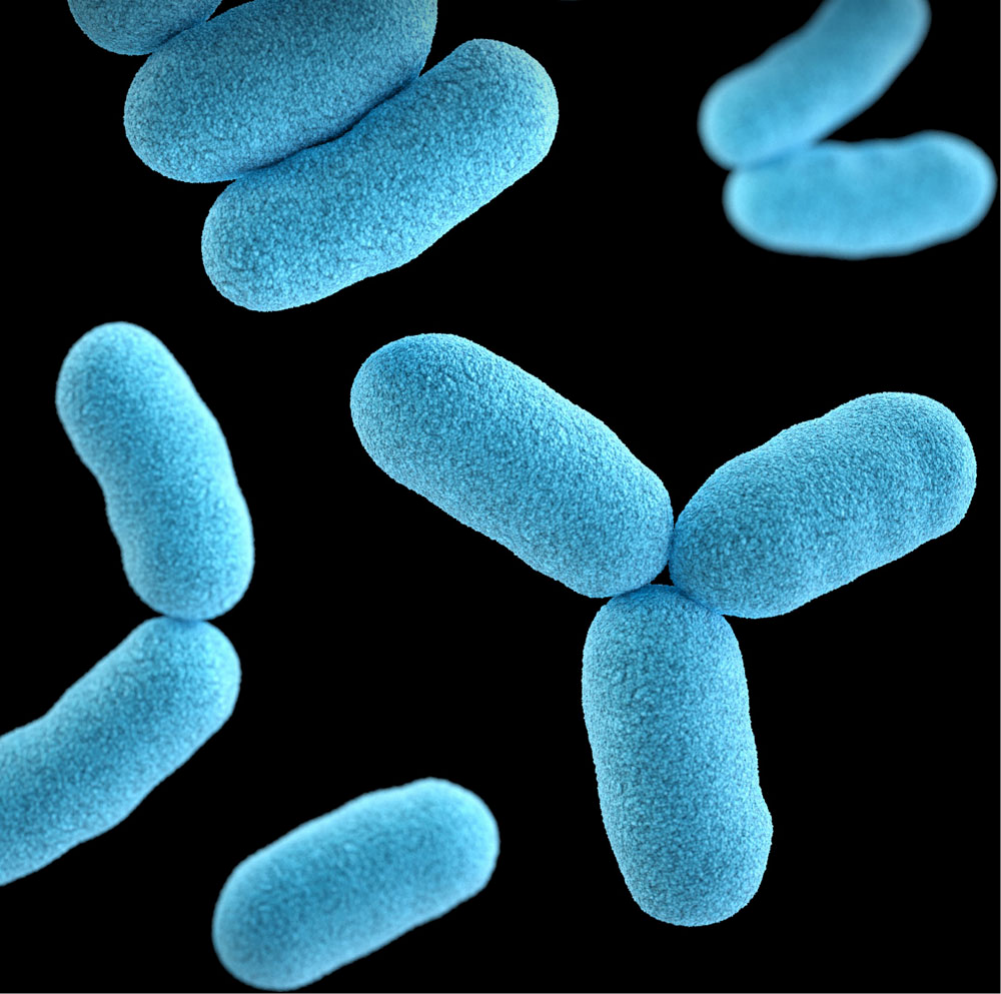
CDC on Unsplash

Certain diseases, such as type 2 diabetes, are known to impact the composition of the gut microbiome—or the community of microorganisms within the gut—as can drugs used to treat disease, including acarbose and other antidiabetic drugs. Conversely, there is also evidence that the microbiome can modulate drug efficacy in diabetes and other diseases, although the mechanisms through which this occurs are poorly defined.

Type 2 diabetes is characterised by an impaired ability to regulate glucose levels in the body. The antidiabetic drug acarbose is ingested orally prior to meals and inactivates enzymes within the digestive system that break down carbohydrates, resulting in reduced levels of glucose released into the bloodstream.

A recent study by Balaich and colleagues found that bacteria within the oral and gut microbiome are able to inhibit acarbose^1^. Via computational analysis of microbiome sequencing data (metagenomics), the authors identified specific bacterial species carrying genes encoding kinases similar to a kinase known to phosphorylate acarbose to generate a product with reduced ability to inhibit carbohydrate-digesting enzymes (AcbK). Acarbose-kinase activity was biochemically demonstrated for a subset of these, designated as Maks.

The most abundant Mak, Mak1, was shown to phosphorylate and inactivate acarbose in a similar way to AcbK and to confer resistance to acarbose when expressed in bacteria. Using metagenomics data from patients on a previous clinical trial, *mak*-positive patients were found to be less responsive to acarbose than *mak*-negative patients.

Further research is needed in larger patient cohorts to evaluate the utility of *mak* genes as biomarkers of acarbose response. However, these findings suggest that the impact of the microbiome on drug response is at least partly due to biochemical products of the microbiome itself, via direct microbiome–drug interaction, and suggest potential opportunities to target the microbiome to improve drug efficacy.

## References

[1] Balaich J (2021). The human microbiome encodes resistance to the antidiabetic drug acarbose. Nature.

